# Hyperspectral remote sensing to detect leafminer‐induced stress in bok choy and spinach according to fertilizer regime and timing

**DOI:** 10.1002/ps.5758

**Published:** 2020-02-07

**Authors:** Hoang DD Nguyen, Christian Nansen

**Affiliations:** ^1^ Department of Entomology and Nematology University of California Davis Davis CA USA

**Keywords:** IPM, machine vision, reflectance profiling, bok choy, spinach

## Abstract

**BACKGROUND:**

Detection and diagnosis of emerging arthropod outbreaks in horticultural glasshouse crops, such as bok choy and spinach, is both important and challenging. A major challenge is to accurately detect and diagnose arthropod outbreaks in growing crops (changes in canopy size, structure, and composition), and when crops are grown under three fertilization regimes. Day‐time remote sensing inside glasshouses is highly sensitive to inconsistent lighting, spectral scattering, and shadows casted by glasshouse structures. To avoid these issues, a unique feature of this study was that hyperspectral remote sensing data were acquired after sunset with an active light source. As part of this study, we describe a comprehensive approach to performance assessment of classification algorithms based on hyperspectral remote sensing data.

**RESULTS:**

Based on average hyperspectral remote sensing profiles from individual crop plants, none of the 31 individual spectral bands showed consistent significant response to leafminer infestation and non‐significant response to fertilizer regime. Multi‐band classification algorithms were subjected to a comprehensive performance assessment to quantify risks of model over‐fitting and low repeatability of classification algorithms. The performance assessment of classification algorithms addresses the important ‘bias‐variance trade‐off’. Truly independent validation (training and validation data sets being separated over time) revealed that leafminer infestation could be detected with >99% accuracy in both bok choy and spinach.

**CONCLUSION:**

We conclude that detailed hyperspectral profiles (not single spectral bands) can accurately detect and diagnose leafminer infestation over time and across fertilizer regimes. Hyperspectral remote sensing data acquisition at night with an active light source has the potential to enable arthropod infestations in glasshouse‐grown crops, such as, bok choy and spinach. In addition, we conclude that effective use and deployment of hyperspectral remote sensing requires thorough performance assessments of classification algorithms, and we propose an analytical performance method to address this important matter. © 2020 The Authors. *Pest Management Science* published by John Wiley & Sons Ltd on behalf of Society of Chemical Industry.

## Graphical abstract

Use of hyperspectral remote sensing to detect and diagnose leafminer‐induced stress across fertilizer regimes and over time.

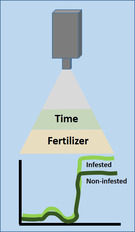

## 1 INTRODUCTION

Inside glasshouses, abiotic conditions (i.e. temperature, light intensity, water availability, nutrient availability) can readily be monitored and controlled to optimize crop growth conditions. However, optimized crop growing conditions inside glasshouses also tend to favor arthropod pest population dynamics. In addition, under glasshouse conditions, rapid increases in arthropod pest populations may be exacerbated by absence (unless released as part of biological control programs) of natural enemies.^1^ Arthropod pest management is therefore a major component of glasshouse operations.^2^, ^3^ Irrespectively of whether biological control programs are in place or not, the favorable abiotic conditions imply that glasshouse pest management must include frequent and reliable monitoring to detect and diagnose emerging arthropod pest outbreaks. As part of integrated pest management programs,^4^, ^5^, ^6^, ^7^ monitoring is a key element that provides knowledge about temporal and spatial changes in arthropod pest population dynamics.^8^ Furthermore, monitoring data may be used in conjunction with action thresholds to determine when and where actions (i.e. insecticide sprays or releases of natural enemies) are needed.^9^

In glasshouse crop production systems, arthropod pest monitoring is often based on a combination of deployment of traps and visual inspection for actual presence of pests or signs and symptoms of infestation.^10^ Regarding trapping, yellow sticky traps are commonly used in glasshouses due to their ability to capture a wide array of pests including thrips, glasshouse whiteflies, leafminers, and winged aphids.^11^, ^12^ Although trapping has been found effective in detection of presence/absence of pests, inconsistency in the relationship between trap captures and pest population densities in many crop–pest systems is a serious challenge that limits the use of trapping data in combination with action thresholds.^13^, ^14^ Furthermore, despite the availability of various trapping designs, there is still a lack of effective traps for pests, such as, two‐spotted spider mites. As a result, visual inspection of crop plants is very important, but it is labor intensive, time‐consuming and costly in large glasshouses.^10^ In addition, accurate and reliable visual inspection of arthropod‐induced damage to horticultural glasshouse crops may be hampered by abiotic and biotic stressors causing similar signs and symptoms and therefore being challenging to distinguish.^15^, ^16^

To overcome the limitations of current arthropod pest monitoring methods, remote sensing technologies have been researched for their capacity to detect and diagnose the presence/absence of biotic stressors in crops.^17^, ^18^, ^19^ Remote sensing technologies are also being widely used for phenotyping of plants.^20^, ^21^, ^22^, ^23^, ^24^, ^25^, ^26^, ^27^ The term, ‘biotic stressor’, refers here to plant stress conditions caused by living pest organisms or infectious agents (i.e. weeds, arthropods, nematodes, bacteria, fungi, viruses).^17^ Within the context of this study, the term biotic stress is used to describe stress conditions specifically caused by American serpentine leafminers [*Liriomyza trifolii* Burgess (Dipteria: Agromyzidae)]. The use of remote sensing to detect and diagnose crop stress hinges on the assumption that when infested, plants elicit structural, physiological, and biochemical responses which, influence the way plant cells absorb and reflect light.^18^, ^28^, ^29^, ^30^ As a consequence, leaf reflectance profiles from healthy (non‐stressed) and stressed plants are assumed to vary, so that unique reflectance features can be identified and used to accurately classify non‐stressed and stressed plants.

Among different sensors being deployed to detect and diagnose biotic stress in crops, hyperspectral remote sensors provide the most detailed spectral response and are therefore more likely to capture subtle plant stress responses.^18^ Hyperspectral remote sensing has been used successfully in a number of pest–crop systems, including: small cabbage white butterfly larvae *Pieris rapae* L. (Lepidoptera: Pieridae)^31^ and aphid *Myzus persicae* Sulzer (Hemiptera: Aphididae)^32^ in cabbage *Brassica oleracea* L. (Brassicales: Brassicaceae); tomato leafminer *Tuta absoluta* Meyrick (Lepidoptera: Gelechiidae) in tomato *Lycopersicon esculentum* Mill (Solanales: Solanaceae);^33^ and two‐spotted spider mites in glasshouse pepper *Capsicum annuum* L. (Solanales: Solanaceae),^34^ and bean *Phaseolus vulgaris* L. (Fabales: Fabaceae).^35^

As reviewed and discussed regarding remote sensing applications in studies of insects,^36^ model over‐fitting and low repeatability of classification algorithms were highlighted as major challenges to the widespread adoption and use of hyperspectral remote sensing in detection and diagnosis of crop stressors. It is therefore paramount that classification algorithms based on hyperspectral remote sensing data are thoroughly validated and that they are subjected to comprehensive performance assessments. As part of such assessments, the concept of ‘bias‐variance trade‐off’ (dilemma) is highly relevant.^37^ The bias‐variance trade‐off is widely known and studied in machine learning applications and supervised classifications, in which ‘Low bias’ may be interpreted as the classification algorithm having a low level of overall simplification (i.e. advanced non‐linear models), so that the algorithm generates model fits to training data with high degree of accuracy. Conversely, a classification algorithm with ‘high bias’ may be considered partially constrained by simplification and therefore unable to fit the training data with high degree of accuracy, which leads to model under‐fitting. Thus to minimize bias, advanced non‐linear models tend to be preferred. In addition, ‘Low variance’ refers to classification algorithm only showing modest responses (in terms of selection of parameters and in coefficient estimates) when developed based on different training data sets. Conversely, ‘high variance’ generally refers to classification algorithms showing excessive sensitivity to small differences among training data sets, which leads to model‐overfitting. Ideally, the goal is to obtain both low bias and low variance, so that classification outcomes are both sensitive enough to detect important differences among similar objects (low bias and avoiding model under‐fitting) and ‘robust’ across data sets (low variance and avoiding model over‐fitting).

In this study, we acquired hyperspectral remote sensing data from two crops, bok choy [*Brassica rapa* L. var Chinensis (Brassicales: Brassicaceae)] and spinach [*Spinacia oleracea* L. ‘Correnta’ (Caryophyllales: Amaranthaceae)], under three fertilization regimes (low, medium, and high) and with/without experimental leafminer infestations. Despite the growing body of hyperspectral remote sensing research of crop plant responses to biotic stresses, only a few studies have been conducted under glasshouse conditions.^38^ Day‐time remote sensing inside glasshouses is highly sensitive to inconsistent lighting, spectral scattering, and shadows casted by glasshouse structures. To avoid these issues, a unique feature of this study was that hyperspectral remote sensing data were acquired after sunset with an active light source. The main study objective was to develop high‐performance classification algorithms for each crop based on average leaf reflectance to accurately detect leafminer infestation over time and across three fertilizer regimes. As part of this main objective, we describe a comprehensive performance assessment of classification algorithms to address the ‘bias‐variance trade‐off’. Finally, the accuracy of classification algorithms was based on truly independent validation. That is for each crop, we collected two independent time series of data, and one was used as training data set and the other as validation data set. Effective pest management programs rely heavily on accurate, reliable, and practically feasible crop monitoring, and there is a dire need for multi‐disciplinary research teams to combine hyperspectral remote sensing technologies, robotics, and machine learning into automated diagnostic crop stress systems with commercial relevance. This study provides a small but important contribution to this effort.

## 2 MATERIALS AND METHODS

### 2.1 Plants and leafminer infestations

Spinach and bok choy were selected as model crops due to their economic importance. In 2004, spinach production accounted for 53 000 acres in the United States with estimated market value of $245.7 million.^39^ Meanwhile, bok choy is considered the most consumed oriental crop with an annual market value between $44–$53 million.^40^ Leafminers were chosen as the pest (biotic stressor), because this species is a major pest in vegetable crop production for both glasshouse and field crops.^41^ Furthermore leafminers possess a wide host range encompassing crop and non‐crop species from many plant families, including: Brassicaceae, Spinaceae, Apiaceae, Solanaceae, Malvaceae, Liliaceae, Fabaceae, Curcurbitaceae, Asteraceae, and Chenopodiaceae.^42^ In bok choy and spinach production systems, leafminers, if left uncontrolled, can cause yield losses exceeding 60%.^41^, ^43^

Spinach and bok choy plants were grown in glasshouse facilities at Orchard Park, University of California, Davis. All plants were maintained in fine‐meshed cages to avoid cross‐infestations (BugDorm‐2120F Insect Rearing Tent, https://shop.bugdorm.com/). Seeds of bok choy and spinach were purchased from Botanical Interests, Inc (Broomfield, CO, USA) and Cornucopia Seeds (6060 Felton, CA, USA), respectively. Bok choy and spinach plants were grown in individual 4‐in pots filled with UC Agronomy soil mix (pumice–sphagnum peat moss–sand–redwood sawdust, 1:1:1:1; 5.23 kg of dolomite per m^3^ of soil mix). Initially, 3–5 seeds were placed in each pot, and thinned after plant emergence, so that each pot contained a single plant.

The following fertilizer composition was supplied through drip irrigation at three controlled rates [electric conductivity, EC, measured in mS/cm^2^ (millisiemens per square centimeter)]: Low (EC = 1), Medium (EC = 2) and High (EC = 3): (values in ppm): nitrogen (N) = 131.5, phosphorus (P) = 40.5, potassium (K) = 180.0, calcium (Ca) = 101.0, magnesium (Mg) = 52.0, sulfur (S) = 68.5, iron (Fe) = 1.5, copper (Cu) = 0.1, manganese (Mn) = 0.3, molybdenum (Mo) = 0.1, and zinc (Zn) = 0.1. For each crop, plants were assigned randomly to fertilization regimes (pots were labeled and assigned to treatments at the onset of seeding). These fertilization regimes were considered to represent a realistic (commercially relevant) range, and each plant was grown under the given fertilization regime from seeding to completion of data collection (5 weeks after seeding).

### 2.2 Experimental design

Data from bok choy and spinach were analyzed separately, and individual plants represented the experimental unit. Both crops were grown in two separate time series in April and May 2018. Replication over time was performed to include stochastic variance associated with experimental growing conditions. That is, even though plants were grown under experimentally controlled conditions, it was considered imperative to replicate data acquisitions over time, so that the potential capability of hyperspectral remote sensing was assessed based on data that encompassed temporal stochasticity in plant growth. In both time series, we maintained bok choy and spinach plants under three continuous fertilizer regimes (see earlier), and bok choy and spinach were randomly divided into two equal groups: non‐infested control plants or plants subjected to leafminer infestations. Infestations were performed 4 weeks after planting and consisted of transferring ten leafminer adults (five males and five females) to individual plants. Leafminer adults used in this study were obtained from a continuous colony maintained at University of California, Davis, CA, USA. Thus, the experimental design comprised three treatment factors: time series (two levels), fertilizer regimes (three levels), and infestation (yes/no). However, we focused exclusively on detection of presence/absence of leafminer infestation within a context that included variance imposed by time, fertilizer regime, and plant ontogeny. We did not consider the severity of leafminer infestations, as we believe that end‐users would be mainly interested in a dichotomous detection algorithm (presence/absence of leafminer infestation).

### 2.3 Hyperspectral remote sensing

**Figure 1:**
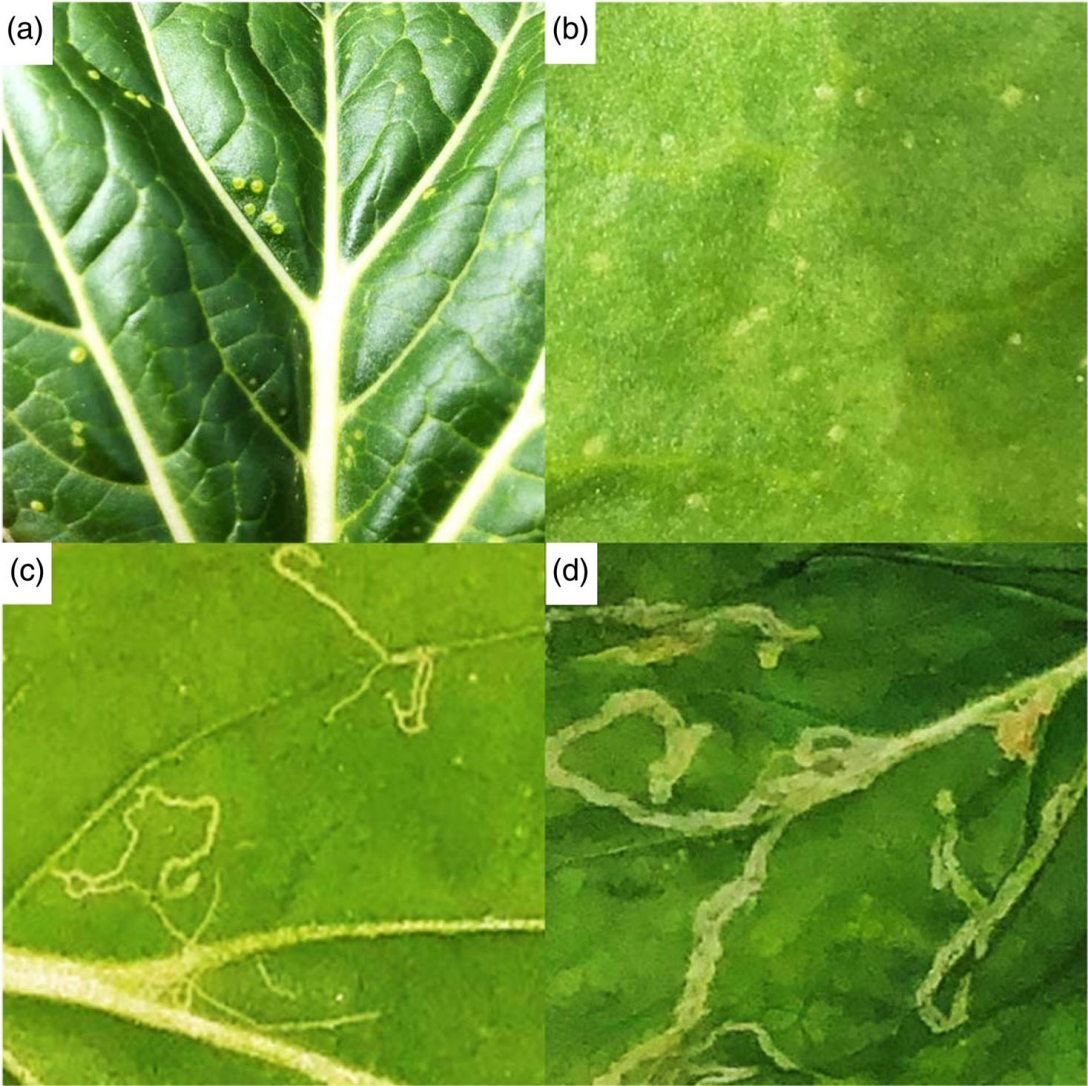
Damage caused by leafminers during different periods of experimental infestation. Leaf damage caused by egg‐laying and feeding leafminer adults during early infestation period in (a) bok choy and (b) spinach. Leaf damage caused by mining activities of newly emerged larvae during late infestation period in (c) bok choy and (d) spinach.

**Figure 2:**
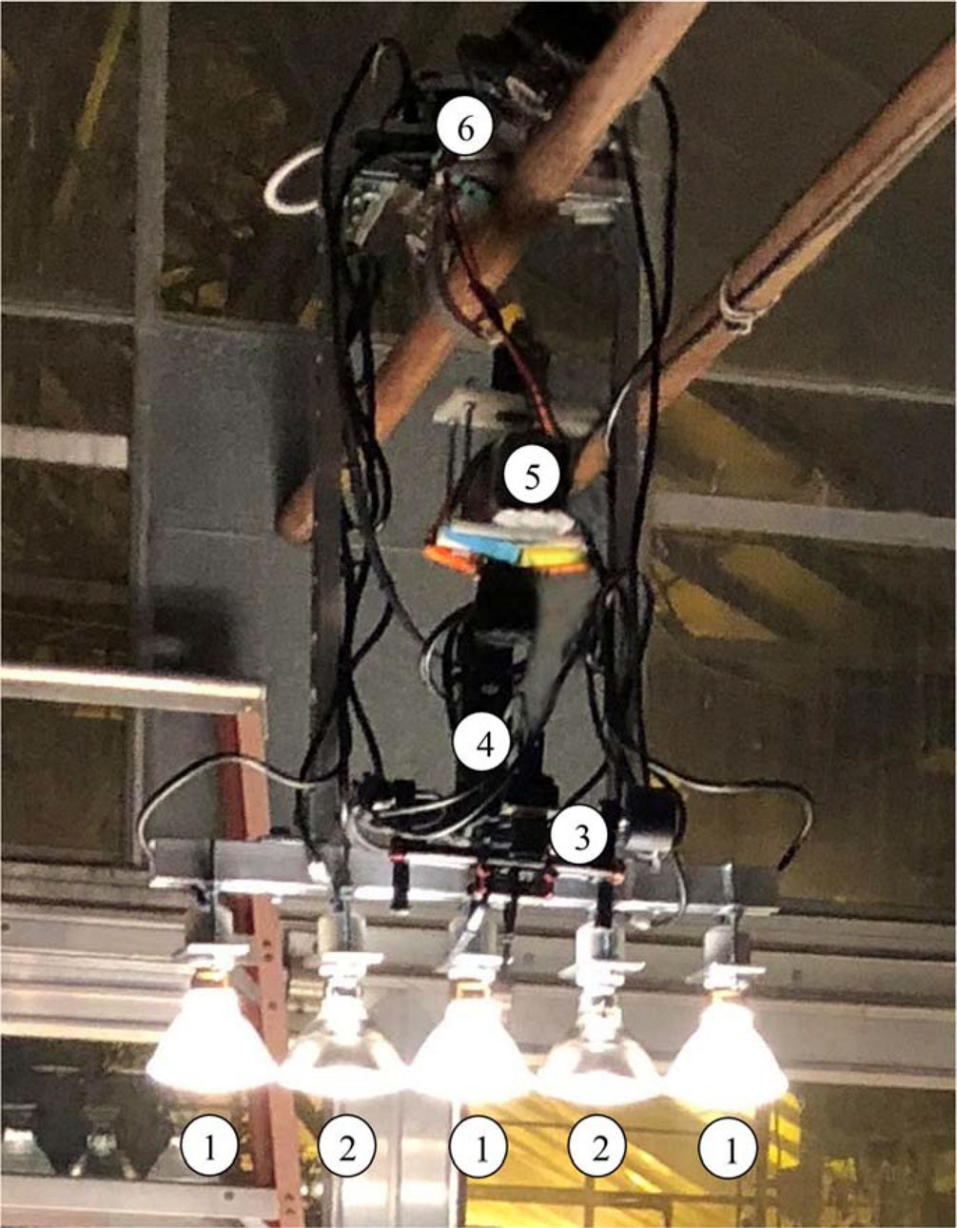
Night‐based hyperspectral remote sensing system. 1, Full‐spectrum light bulbs (PLT‐11088 250 W – PAR38 – Flood – Halogen – 3750 Lumens – 120 V); 2, infra‐red light bulbs (PAR‐138628 90 W – PAR38 – Narrow Flood – IR Halogen – 1550 Lumens); 3, BaySpec hyperspectral camera; 4, remotely controlled DJI Ronin‐M gimbal; 5, portable battery to power remotely controlled motor; 6, remotely controlled motor mounted with portable computer hard drive to store data and connect hyperspectral camera with remote control laptop.

Hyperspectral remote sensing data were acquired from crop plants in time series, comprising: baseline (before infestation), 2, 4, 7, 8 and 10 days of leafminer infestation. Within the first 4 days of infestation, the only sign of leaf damage was induced by feeding and egg‐laying leafminer adults (Fig. 1(a, b)). Mining activities caused by newly emerged larvae could be clearly observed from day seven and onwards (Fig. 1(c, d)). Consequently, we combined hyperspectral remote sensing data into three time periods: baseline, early (2–4 days), and late (7–10 days) of leafminer infestation. Hyperspectral remote sensing data were acquired from 9:00–11:00 p.m. using a controlled lighting system comprising a mixture of three full‐spectrum halogen and two infra‐red halogen light bulbs in a single line that was moving together with the hyperspectral remote sensors to ensure constant lighting (Fig. 2).

We collected hyperspectral imaging data with an imaging sensor [OCI Imager (OCI‐UAV‐D1000), BaySpec Inc., San Jose, USA], which acquires reflectance data in 116 spectral bands between 460 and 983 nm. However, due to high levels of stochastic noise, we only included reflectance data from 93 spectral bands between 619.5 nm and 983.0 nm. We performed 3× spectral binning (averaging of spectral bands) of the 93 spectral bands, so that 31 spectral bands were used in analyses. Spectral binning serves three important and highly complementary purposes: (i) in some cases, it has been shown to increase classification accuracy (likely due to data smoothening),^44^ (ii) it reduces the size of input data sets and therefore increase the data processing speed, and (iii) it minimizes the risks of model‐overfitting caused by the Hughes phenomenon or violation of the principle of parsimony.^45^, ^46^ White calibration was done by obtaining reflectance data from white Teflon board. To exclude background pixels (ground, pots, soil, etc.), we used a previously published radiometric filter by Rouse *et al*.,^47^ in which only pixels within specific Normalized Difference Vegetation Index (NDVI) were included: 0.54 < NDVI <0.85 for spinach and 0.55 < NDVI <0.70 for bok choy. After radiometric filtering and white calibration, reflectance values of all pixels acquired from individual plants were averaged to obtain an average reflectance profile from each plant.

### 2.4 Data analysis

All data processing steps and analyses of hyperspectral remote sensing data were performed using the statistical software, R v3.61 (The R Foundation for Statistical Computing, Vienna, Austria). All data analyses were based on first derivative spectra calculated using the method described in Mercer.^48^ In all classifications, average leaf reflectance data from individual plants were analyzed. Initially, we use the lme4 library in R to conduct linear mixed model analyses of average spectral derivatives in each of the 31 spectral bands with fertilizer regime and leafminer infestation as treatment factors and with time series of data sets as random variable. We conducted separate linear mixed model analyses for each of the three infestation periods (baseline, early and late infestation) and each crop (bok choy and spinach). Secondly, we used the library e1071 in R to perform support vector machine classifications (kernel = ‘linear’) of each crop, in which average reflectance values in the 31 spectral bands were used as explanatory variables. Support vector machine is a widely used machine learning algorithm, and it is commonly used in classifications of remote sensing data.^49^ An important feature of support vector machine is that these algorithms are generally considered to possess low bias and low variance and therefore provide high levels of both robustness and sensitivity.^49^ In each classification algorithm (one for bok choy and one for spinach), average reflectance profiles from individual plants from each crop (observations) were assigned to one of 15 classes, which represented all combinations of the three factors, leafminer infestation, time period (no infestation with leafminer for time period = baseline), and fertilizer regime. An example of one of the classes would be: leafminer infestation = 1, time period = 1, and fertilizer regime = 1. Regarding the 15 classes: six classes represented leafminer infestation under different fertilizer regimes and at different time points, and the remaining nine classes represented non‐infested classes (baseline data represented the three extra classes of non‐infested plants). Thus, if a non‐infested plant from time period 1 was classified as a non‐infested plant from time period 2, the classification algorithm would consider that a ‘mis‐classification’ (because the observation was placed in the wrong time period class). However, the main goal of our classification algorithms was not to predict the age of plants and/or the fertilizer regime, but rather to accurately diagnose crop plants are infested with leafminer or not. Thus, we corrected for ‘mis‐classifications’ associated with non‐infested or infested plants being correctly classified as non‐infested or infested but assigned to the incorrect time period and fertilizer regime.

As a performance assessment to examine the bias‐variance trade‐off of each of the two classification algorithms, we performed a series of cross‐validations by partitioning the entire data set into different fractions of training and validation data. That is, we randomly selected 50–95% of entire data set as training and the remaining data (5–50%) were used validation data. For instance, if 70% of the entire data was used as training data, the remaining 30% of the data were used as validation data. This procedure was repeated with 1000 random partitions for each of six partition levels of training and validation data (50:50, 60:40, 70:30, 80:20, 90:10, and 95:5). Frequency distributions of the 1000 classifications for each data partition were examined and used to discuss the performance of each classification algorithm.

As a final evaluation of the two classification algorithms, we conducted truly independent validation. Moreover for each crop, hyperspectral remote sensing data had been collected in two separate time series, and we therefore used data from one time series as training data and the second time series data for validation. And this was performed with both time series as either training or validation data sets.

## 3 RESULTS

### 3.1 Use of individual spectral bands to detect leafminer infestation

**Figure 3:**
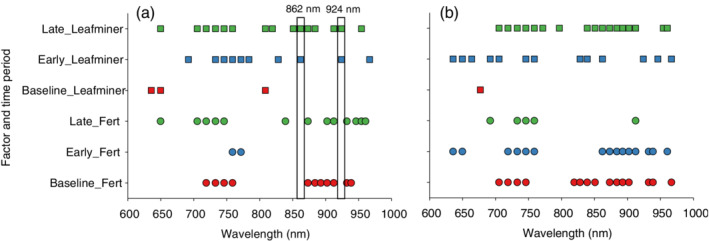
Analyses of variance of average first derivatives in individual spectral bands. Only statistically significant (*P* < 0.05) effects of leafminer infestation (squares) and fertilizer regime (filled dots) of bok choy (a) and spinach (b) are presented. Separate analyses of variance were performed for three time periods [baseline (before infestation) are presented as red symbols, ‘early’ (2–4 days of infestation) are presented as blue symbols, and ‘late’ (7–10 days of infestation) are presented as green symbols]. In (a) spectral bands 862 and 924 nm are highlighted, because they showed significant responses to leafminer infestation without showing significant response to fertilizer regime.

The main purpose of the linear mixed model analyses was examine to what extent individual spectral bands responded significantly to leafminer infestation without showing a significant response to fertilizer regimes. Such results would support the claim that individual spectral bands may be used to identify leafminer‐induced stress, even when examined among plants subjected to different fertilizer regimes. With 31 spectral bands and three time intervals, we analyzed responses to fertilizer regime and leafminer infestation in all 93 combinations. In analyses of bok choy (Fig. 3(a)), about one‐third of the combinations showed significant responses to fertilizer regime or leafminer infestation, and only a single spectral band (759 nm) responded significantly to leafminer infestation in all three time intervals. However, the same spectral band also responded significantly to fertilizer regime and could therefore not be considered a reliable indicator of leafminer infestation. In analyses of spinach, more combinations (leafminer = 31 and fertilizer regime = 38) responded to fertilizer regime than to leafminer infestation (Fig. 3(b)). Several spectral bands responded significantly to leafminer infestation in all three time intervals, but they also responded significantly to fertilizer regime. Thus, these analyses of average responses in individual spectral bands to fertilizer regimes, periods of infestation, and leafminer infestation provided strong support for the claim that responses to specific biotic stressors (in this case leafminer infestation) may vary among crop plants. In addition, the current analysis suggests that accurate diagnosis of stress responses to a specific biotic stressor should be based on multivariate classifications of reflectance values in spectral bands.

### 3.2 Multivariate classification to detect leafminer infestation

**Figure 4:**
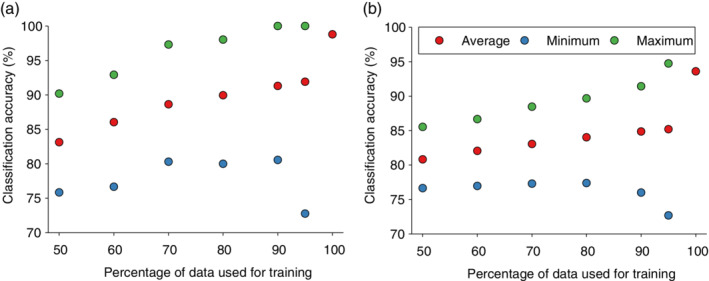
Classification accuracies (minimum, maximum, and average) of leafminer infestation. Entire data sets acquired from bok choy (a) and spinach (b) were portioned into training and validation data with training data accounting for 50 to 95% of the data. For each training: validation data ratio, 1000 randomized partitions were performed, and minimum, maximum, and average classification accuracies were determined.

Using the entire data set acquired from bok choy (*N* = 344) as training data (no validation), leafminer infestation was classified with 98.8% accuracy, as three control plants were mis‐classified as infested plants. Using the entire data set acquired from spinach (*N* = 348), leafminer infestation was classified with 98.3% accuracy, as two control plants were mis‐classified as infested plants, and four infested plants were mis‐classified as control plants. Although these classification results strongly suggest that leafminer infestation can be detected consistently across a range of fertilizer regimes, it is paramount to investigate classification accuracies after partitioning of the entire data into model training and the remaining data being used for validation. In this study, we performed 1000 iterations of data partitions (training and validation data) with 50–95%, and we focused exclusively on the ability to accurately classify crop plants with/without leafminer infestation. Figure 4(a) shows that minimum classification accuracies of leafminer in bok choy exceeded 75% for all data partitions, except when 95% of the entire data was used as training data. Moreover, small validation data sets lead to higher contribution of each observation in the calculation of classification accuracies and therefore an increase in likelihood of obtaining a lower classification accuracy. Thus, it is basically an artifact caused by small data set size. Figure 4(a) also shows that the average classification accuracy increased gradually from about 83% to 90% as a function of adding to the relative size of the training data set. Figure 4(b) shows that the average classification accuracy of leafminer in spinach was similar to that in bok choy, and it exceeded 80% for all data partitions.

Table 1 shows the average classification results from true validation, when one time series data set was used as training data and the other used as validation data. In the validation of hyperspectral remote sensing based classification of bok choy plants, the two time series data sets contained 171 and 173 average profiles, respectively. Of these 344 average profiles from bok choy plants, three were mis‐classified as infested with leafminers (false positive), while none were mis‐classified as non‐infested (false negative). All other mis‐classifications represented inaccurate prediction of age of plant and/or of fertilizer regime. Thus based on true validation, the accuracy of leafminer infestation was 341/344 = 99.1%. In the validation of hyperspectral remote sensing based classification of spinach plants, the two time series data sets contained 170 and 178 average profiles, respectively. Of these 348 average profiles from spinach plants, two were mis‐classified as infested with leafminers (false positive) and one plant was mis‐classified as non‐infested (false negative). All other mis‐classifications represented inaccurate prediction of age of plant and/or of fertilizer regime. Thus based on true validation, the accuracy of leafminer infestation was 345/348 = 99.1%.

**Table 1:** Classification results based on true validation

Code	Time	Fert	Inf	1	2	3	4	5	6	7	8	9	10	11	12	13	14	15	Plants	
Bok choy																				
1	Base	Low	No	90.0	2.5	2.5	2.5		2.5										20	
2	Base	Med	No	7.5	82.5	7.5			2.5										20	
3	Base	High	No	10.0	7.5	82.5													20	
4	Early	Low	No	16.7			77.8			**5.6**									9	
5	Early	Med	No	5.0		5.0		78.9	5.6		**5.6**								9.5	
6	Early	High	No			5.6			88.9	**5.6**									9	
7	Early	Low	Yes							83.3	11.1	5.6							9	
8	Early	Med	Yes							18.1	75.7	6.3							8.5	
9	Early	High	Yes							5.6		94.4							8	
10	Late	Low	No										100.0						10	
11	Late	Med	No											100.0					10	
12	Late	High	No												100.0				10	
13	Late	Low	Yes													89.4	5.0	5.6	9.5	
14	Late	Med	Yes													5.0	95.0		9.5	
15	Late	High	Yes													5.0	20.0	75.0	10
Spinach																			
1	Base	Low	No	95.0		5.0													10
2	Base	Med	No		50.0	50.0													10
3	Base	High	No			100.0													40
4	Early	Low	No			5.0	95.0												10
5	Early	Med	No			15.0	5.0	75.0	5.0										10
6	Early	High	No			10.0			79.4		**10.6**								9.5
7	Early	Low	Yes							95.0	5.0								10
8	Early	Med	Yes								90.0	10.0							10
9	Early	High	Yes								5.6	94.4							9
10	Late	Low	No										100.0						10
11	Late	Med	No											100.0					10	
12	Late	High	No												95.0		5.0		9	
13	Late	Low	Yes													100.0			9	
14	Late	Med	Yes												**5.0**		88.8	6.3	9	
15	Late	High	Yes													6.3	5.6	88.2	8.5	

The column, ‘Code’ denotes the actual treatment classes. ‘Time’ denotes the three time periods: baseline, early (2–4 days), and late (7–10 days) of leafminer infestation. ‘Fert’ denotes fertilizer regime (low, medium, and high). ‘Inf’ denotes leafminer infestation (Yes or No). Columns 1–15 denotes codes of predicted treatment classes, and values in these columns represent percentages of plants assigned to that class. Percentages in bold represent mis‐classifications of leafminer infestation, and bordered and bold percentages represent false negative mis‐classifications. ‘Plants’ denotes the average number of crop plants for the given treatment class. Treatment classes representing leafminer infestation are highlighted in gray.

## 4 DISCUSSION

Very few studies have examined crop leaf reflectance responses to abiotic and biotic stressors simultaneously. Detection of one or several stressors within a context of multiple agronomic, environmental, and biological factors represents a major challenge but it is also the only way hyperspectral remote sensing studies can lead to the development of diagnostic crop stress systems with commercial relevance. The main purpose of this study was to use leaf reflectance data to diagnose leafminer infestation in bok choy and spinach across fertilization regimes and over time. We demonstrated that individual spectral bands may respond significantly to leafminer infestation but they also responded significantly to fertilizer regimes. Thus, individual spectral bands were considered unsuitable for reliable detectors of arthropod stress. For comparison, support vector machine classifications of average leaf reflectance profiles in 31 spectral bands provided high‐accuracy and high‐consistency detection of leafminer infestation in both bok choy and spinach. Truly independent validation (training and validation data sets being separated over time) revealed that leafminer infestation could be detected with > 99% accuracy in both bok choy and spinach. It is very important to emphasize that the obtained classification accuracies of leafminer infestation were generated from data sets, in which plants were grown in different time series, under three different fertilizer regimes, and with plants varying in age/ontogeny.

Individual spectral bands near 700 nm, 800 nm and 900 nm have been found highly correlated with plant responses to biotic stressors.^10^ Furthermore, leaf reflectance near these spectral bands have been widely used to construct various vegetation indices for biotic stress detection, including NDVI,^47^ Red Edge Position (REP),^50^ Structure Insensitive Vegetation Index (SIVI),^51^ Green Normalized Difference Vegetation Index (GNDVI),^52^ Optimized Soil Adjusted Vegetation Index (OSAVI),^53^ and Aphid Index.^54^ The physiological mechanism behind the use of leaf reflectance near 700 nm is believed to involve changes in chlorophyll a and b in leaf tissues, when plants are under biotic stress.^55^, ^56^, ^57^, ^58^ Meanwhile, leaf reflectance near 800 nm is associated with leaf pigments and internal structure.^59^ Furthermore, leaf reflectance near 900 nm has been shown to be related to water absorption and indicative of leaf water content when plants are under biotic stress.^60^, ^61^, ^62^ We found no evidence of individual spectral bands responding consistently to leafminer infestation in both bok choy and spinach. Thus, we provided convincing support for the claim that detection methods based on single spectral bands (and possible also simple spectral indices) may have limited use, when remote sensing data are acquired from plants grown under varying abiotic conditions and with remote sensing data being acquired over time. Nansen *et al*.^38^ identified five spectral bands (440, 462, 652, 706, and 784 nm) that could be used to differentiate corn plant subjected to different irrigation regimes (moderate, subtle, and none), across hybrids, leaf position on plants, and presence/absence of spider mite infestation. Susič *et al*.^63^ used examined leaf reflectance data from tomato plants subjected to combinations of drought stress and infestation by root‐knot nematode *Meloidogyne incognita* (Tylenchida: Heteroderidae) (biotic). The study found spectral regions around 842–857 nm and 944–958 nm uniquely sensitive to root‐knot nematode infestation with classification accuracy ranging from 90 to 100%.

In data sets for both crops, the ratio between observations (number of average reflectance profiles) and number of explanatory variables exceeded ten. That is, there were > ten times more observations (bok choy = 344 and spinach = 348 observations) than explanatory variables (31 spectral bands + time period and fertilizer regime = 33 explanatory variables). Consequently from a data structure standpoint, the risk of model over‐fitting (high variance) was very low, as the general recommendations to minimize risk of model over‐fitting (high variance) is to have at least twice as many observations as explanatory variables.^36^, ^44^, ^45^, ^46^ Support vector machine algorithms are considered highly suitable for classifications of remote sensing data, and they provide optimized/balanced relationship between model fitting to training data set (bias) and ability to accurately classify validation data (variance).^49^ Using support vector machine, we developed classification algorithms, which detected leafminer infestation with accuracies exceeding 99% in both bok choy and spinach. Several studies have demonstrated successful use of support vector machine algorithms for detection of biotic stress in crops.^49^, ^64^

Numerous studies describe and discuss important aspects of hardware and practical challenges associated with high‐throughput remote sensing of plants.^26^, ^65^ The results from this study provided substantial support for the claim that night‐based hyperspectral remote sensing can be used in crop monitoring, and pest outbreak detection in particular. If so, the implications for integrated pest management are considerable. Moreover, development of biotic stress detection in glasshouse crops based on hyperspectral remote sensing may enhance the ability to detect pest outbreaks early and therefore lead to: (1) spot‐treatments (in contrast to broadcast) with insecticides and therefore less overall use of insecticides, (2) spot releases of natural enemies, and that may promote biological control programs, which may be cost‐prohibitive and practically unfeasible to implement on a large scale.

Hyperspectral remote sensing studies, combined with thorough performance assessments of classification algorithms, are needed when the ultimate goal is to combine hyperspectral remote sensing technologies, robotics, and machine learning into overhead rail or autonomous rover‐based systems to detect and diagnose crop stress systems under commercial settings. For the full potential of hyperspectral remote sensing to be explored to detect and diagnose biotic stressors in glasshouse crops, it is important to highlight that development of classification algorithms is just one of several important elements. There are numerous challenges associated with robotics (hardware performance) and image data processing (transfer, storage and classification). Thus if the ultimate goal is to develop commercial solutions that enable precision pest management based on hyperspectral remote sensing, there is a high demand and need for applied multi‐disciplinary research collaborations, involving entomologists, agronomists, software engineers, optical physicists, electrical engineers, mechanical engineers, and private sector partners.
